# National Conference of State Medical Examining Boards

**Published:** 1891-05

**Authors:** 


### NATIONAL CONFERENCE OF STATE MEDICAL EXAMINING BOARDS.

Dr. John H. Rauch, Secretary of the Illinois State Board, has, at the suggestion of Dr. William Perry Watson, Secretary of the New Jersey Board, issued a letter of invitation for representatives of the several State Examining and Licensing Boards to meet in Washington, D. C., May 6, 1891, during the meeting of the American Medical Association. It is proposed at the meeting to effect a permanent organization, consider rules relating to uniformity of practice in the conduct of the examinations, and to transact such other business as may come before the conference. The suggestion seems to be a good one, and it is presumed there will be a full attendance of representatives of the twenty-one State Boards now in existence.

